# Association of reproductive factors and exogenous hormone use with distal sensory polyneuropathy among postmenopausal women in the United States: results from 1999 to 2004 NHANES

**DOI:** 10.1038/s41598-023-35934-7

**Published:** 2023-06-07

**Authors:** Jiayu Li, Yuda Chongpison, Jakkrit Amornvit, Sukanya Chaikittisilpa, Somsook Santibenchakul, Unnop Jaisamrarn

**Affiliations:** 1 Department of Obstetrics and Gynecology, Faculty of Medicine, Chulalongkorn University, Bangkok, Thailand; 2 Center of Excellence in Biostatistics, Research Affairs, Faculty of Medicine, Chulalongkorn University, Bangkok, Thailand; 3 The Skin and Allergy Research Unit, Faculty of Medicine, Chulalongkorn University, Bangkok, Thailand; 4 Division of Neurology, Department of Medicine, Faculty of Medicine, Chulalongkorn University, Bangkok, Thailand; 5 King Chulalongkorn Memorial Hospital, Thai Red Cross Society, Bangkok, Thailand; 6 Menopause Research Group, Department of Obstetrics and Gynecology, Faculty of Medicine, Chulalongkorn University, Bangkok, Thailand; 7 Family Planning and Reproductive Health Unit, Department of Obstetrics and Gynecology, Faculty of Medicine, Chulalongkorn University, Rama 4 Road, Bangkok, 10330 Thailand

**Keywords:** Neurological disorders, Reproductive disorders, Risk factors

## Abstract

Postmenopausal status is a risk factor for distal sensory polyneuropathy—the most common type of peripheral neuropathy. We aimed to investigate associations between reproductive factors and history of exogenous hormone use with distal sensory polyneuropathy among postmenopausal women in the United States using data from the National Health and Nutrition Examination Survey 1999–2004, and to explore the modifying effects of ethnicity on these associations. We conducted a cross-sectional study among postmenopausal women aged ≥ 40 years. Women with a history of diabetes, stroke, cancer, cardiovascular disease, thyroid disease, liver disease, weak or failing kidneys, or amputation were excluded. Distal sensory polyneuropathy was measured using a 10-g monofilament test, and a questionnaire was used to collect data on reproductive history. Multivariable survey logistic regression was used to test the association between reproductive history variables and distal sensory polyneuropathy. In total, 1144 postmenopausal women aged ≥ 40 years were included. The adjusted odds ratios were 8.13 [95% confidence interval (CI) 1.24–53.28] and 3.18 (95% CI 1.32–7.68) for age at menarche < 11 years and time since menopause > 20 years, respectively, which were positively associated with distal sensory polyneuropathy; adjusted odds ratios were 0.45 for the history of breastfeeding (95% CI 0.21–0.99) and 0.41 for exogenous hormone use (95% CI 0.19–0.87) were negatively associated. Subgroup analysis revealed ethnicity-based heterogeneity in these associations. Age at menarche, time since menopause, breastfeeding, and exogenous hormone use were associated with distal sensory polyneuropathy. Ethnicity significantly modified these associations.

## Introduction

Distal sensory polyneuropathy (DSP) is a neurological disorder that is commonly encountered by family physicians and neurologists. It is characterized by symmetric distal foot or toe numbness, tingling, with/without neuropathic pain, or loss of sensation^1^. Some patients may only experience mild paresthesia or negative symptoms; however, it is challenging to reverse established neuropathy^2^. Moreover, ignoring these signs could eventually put patients at risk for various diseases or may be life-threatening^3^. Approximately 28% of American adults with diabetes suffer from DSP, which accounts for approximately $10.91 billion in healthcare expenditure in the United States (US)^4–6^. Among the non-diabetic population, prediabetes and metabolic syndrome components, such as obesity and hypertension, have been reported as common causes of cryptogenic sensory peripheral neuropathy^2^. In addition, cytostatic drugs, nutritional deficiency, alcoholism, smoking, and cardiovascular disease could also result in DSP^7–9^. Nevertheless, sometimes the neurologist is unable confirm the particular cause despite careful history and exclusive assessment^10^. Approximately 5–8 million Americans are affected by idiopathic (unknown cause) peripheral neuropathy^10^.

Several studies reported that women exhibited a 1.5 to twofold greater risk of DSP than men^9^. The sex differences in the prevalence of peripheral neuropathy may be due to women's longer lifespan and the reduced estrogen levels following menstrual transition among postmenopausal women^11–13^. The prevalence of DSP among Egyptian women was reported to peak in the 45–54 age group^14^. Women undergo menopause around this age, ending their reproductive phase^15^. Postmenopausal status has consistently been a risk factor for polyneuropathy in non-diabetic obese individuals^16^. Peripheral neuropathy always coexists with central nervous system diseases^17,18^. The widely fluctuating hormone levels during each reproductive stage and the cumulative lifetime estrogen exposure have a substantial impact on women’s central nervous system later in life^13^. Moreover, aging nerves are sensitive to exogenous estrogen in animal studies^19,20^. Nevertheless, there are no reports in the literature regarding whether reproductive factors and a history of exogenous hormone use are associated with the prevalence of peripheral neuropathy among postmenopausal women.

This cross-sectional study was designed to explore the associations between DSP and reproductive history factors, including age at menarche, pregnancy, breastfeeding, age at menopause, total reproductive lifespan, time since menopause, and history of exogenous hormone use among non-diabetic postmenopausal women using publicly published data from the National Health and Nutrition Examination Survey (NHANES) 1999–2004. As the prevalence of PN was reported to be different by racial/ethnic groups^21^, we also explored the modifying effect of ethnicity of these associations.

## Materials and methods

### Study participants

**Figure 1:**
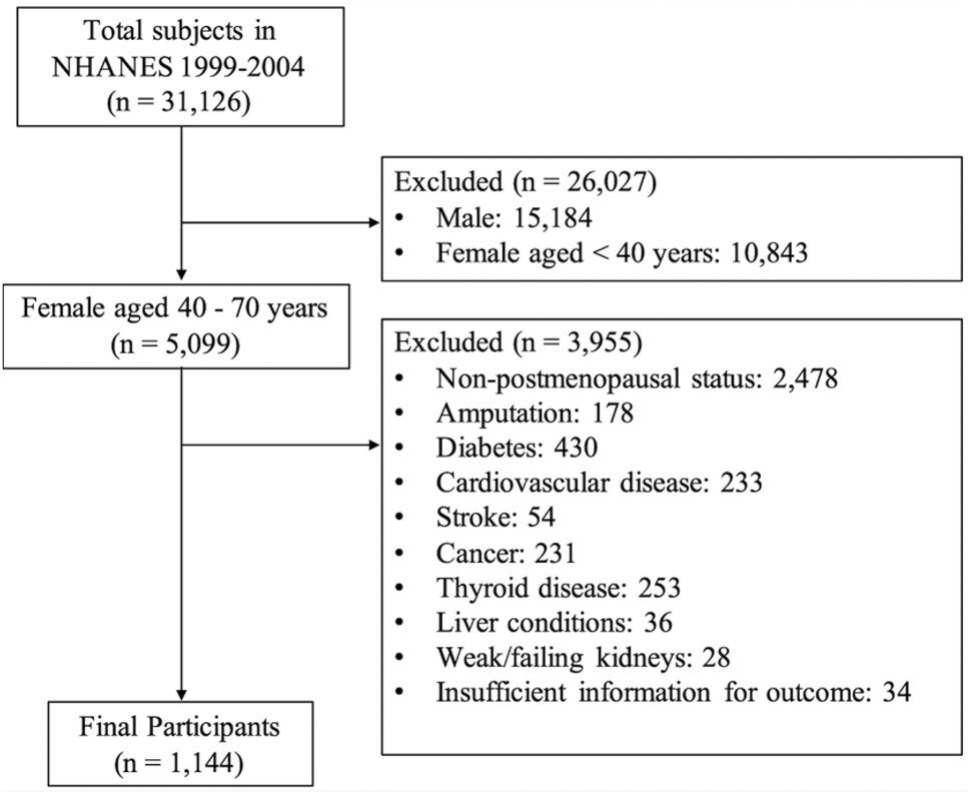
Flow diagram for eligible participants.

We extracted the data of postmenopausal women aged ≥ 40 years from the NHANES 1999–2004^22^. Women with self-reported medical history of diabetes, stroke, cancer, cardiovascular disease, thyroid disease, liver disease, and weak/failing kidneys were excluded, because these diseases are common causes of peripheral neuropathy^3^. Women with amputation or insufficient information regarding their eligibility and outcome were also excluded. Finally, 1144 eligible postmenopausal women were included in the study (Fig. 1). Our study followed the reporting guidelines of the RECORD statement^23^.

The protocols of NHANES were approved by the Institutional Review Board of the Centers for Disease Control and Prevention, and all participants provided informed consent. We downloaded, generated, and cleaned the data from different NHANES cycles to build a dataset for this study. Our study was exempted by the Institutional Review Board of the Faculty of Medicine, Chulalongkorn University (No. 0422/65). All research was performed in accordance with the Declaration of Helsinki. Data from NHANES could be downloaded online. The datasets generated and analyzed during the current study are available from the first author.

### Outcomes

DSP was identified as possessing at least one insensate site on either foot by a 10-g monofilament test, a binary variable: DSP and non-DSP^24^. Participants were asked to lie on the exam table to undergo the sensation of the touch test. Well-trained technicians applied slight pressure to the plantar-first metatarsal head, plantar-fifth metatarsal head, and plantar-hallux of each participant’s foot, without sequential order. An insensate site was defined when participants were unable to respond correctly to the filament pressure. The total number of insensate areas ranged from zero to six for both feet. DSP was defined as ≥ 1 insensate area, which has been reported as a significant predictor for ulcers and amputation, with moderately high sensitivity (∼85%) and specificity (∼80%)^25^.

### Exposure

Self-reported reproductive factors and history of exogenous hormone use were analyzed in this study. Age at menarche was defined as the first menstrual period (≤ 11 and > 11 years). Gravidity was defined as the total number of pregnancies, including current pregnancies, live births, miscarriages, stillbirths, tubal pregnancies, and abortions (< 4 or ≥ 4). Breastfeeding was defined as having ever breastfed by any of their children (never or ever). Postmenopausal status was defined as both ovaries removed (representing the surgical menopause) or having last period at 12 or more months ago with the reason of “going-gone through menopause (representing natural menopause). Age at menopause was defined as the age at the last menstrual period (< 45; 46–55; ≥ 56 years). The total reproductive lifespan was defined as the time from the onset of menarche to the onset of menopause (≤ 35 and > 35 years). Time since menopause was defined as the duration between the age at interview and the age at last menstrual period (≤ 20 and > 20 years). A history of exogenous hormone use was defined as the use of contraceptives, including birth control pills and contraceptive injection, or any type of menopause hormone therapy, including pills, cream, patches, and injectables (never or ever).

### Covariates

The demographic characteristics included age (40–70 and > 70 years), ethnicity (Mexican American and other Hispanic as Hispanic, and Non-Hispanic White, Black and Others as Non-Hispanic), education (less than high school and high school or above), income (prescribed investor rate [PIR] ≤ 2.00 and > 2.00, the sample median cutoff value for PIR is 2), and insurance (covered and not covered). Health-related lifestyle variables included smoking (never or ever) and alcohol consumption (never or ever). Smoking was defined as smoking at least 100 cigarettes in life or being a current smoker at the time of interview. Alcohol use was defined as at least 12 drinks of any type of alcohol throughout life (one drink was 2 oz. beer, a 4 oz. glass of wine, or an ounce of liquor). Health condition-related variables, including hypertension and body mass index (BMI), were also evaluated. Hypertension (yes or no) was defined as the presence of at least one of the following conditions: an average systolic blood pressure ≥ 140 mmHg, an average diastolic blood pressure ≥ 90 mmHg, or self-reported current use of prescribed antihypertensive medication^22^. BMI was calculated as weight in kilograms divided by height in meters squared: underweight/normal (< 25 kg/m^2^) and overweight/obese (≥ 25 kg/m^2^)^26^.

### Statistical analysis

The missing values for each variable in the final analysis are shown in Table 1. Codebook of each variable could be found from ﻿Supplementary Table S1. We used the complete case analysis after data management and cleansing. Proportions with 95% confidence interval (CI) and medians with interquartile ranges (Q1 and Q3) were reported for categorical and numerical data, respectively. Binary logistic regression was conducted to determine the association between reproductive factors and exogenous hormone use with DSP, using the purposeful selection of covariate strategy^27,28^. The significant interaction term of ethnicity and menarche (*p* = 0.03) was fitted into the final multivariable logistic regression model based on both clinical and statistical considerations. We investigated whether ethnicity modifies the association between reproductive factors and DSP using the final multivariable model in the primary analysis (except for ethnicity and the interaction term) for subgroup analysis. Multicollinearity and goodness-of-fit were evaluated for each model. The results are presented as odds ratios (ORs) with 95% confidence intervals (CIs). Statistical significance was set at a two-sided *p*-value < 0.05. Survey weights, clusters, and strata were considered to account for the complex survey design in all statistical analyses using Stata software version 15.1.

**Table 1:** Missingness of all variables (NHANES 1999–2004 cycle, N = 1144).

Variables	n	%
Distal sensory polyneuropathy	0	0.00
Age at menarche	14	1.22
Number of pregnancies	103	9.00
Breastfeeding history	131	11.45
Age at menopause	5	0.44
Time since menopause	71	6.21
Total reproductive life	93	8.13
History of exogenous hormone use	2	0.17
Age	0	0.00
Ethnicity	0	0.00
Education	2	0.17
Income	116	10.14
Alcohol use	1	0.09
Smoking	2	0.17
Hypertension	21	1.84
Body mass index	21	1.84
Insurance	11	0.96

Values are number (n) and percentage (%).

### Ethics declarations

NHANES is a publicly released de-identified dataset. The protocols of NHANES were approved by the Institutional Review Board of the Centers for Disease Control and Prevention, and all participants provided informed consent. We downloaded, generated, and cleaned the data from different NHANES cycles to build a dataset for this study. Our study was exempted by the Institutional Review Board of the Faculty of Medicine, Chulalongkorn University (No. 0422/65). All research was performed in accordance with the Declaration of Helsinki.

## Results

### Participant characteristics

The characteristics of all participants are summarized as weighted prevalence estimates in Table 2. A total of 1144 participants were included in the final dataset: 390 individuals from 1999 to 2000, 387 from 2002 to 2003, and 367 from the 2003–2004 NHANES cycles. Of these, 89 (6.26%) patients were diagnosed with DSP. The median age (Q1, Q3) was 63 (55–73) years, and 798 (77.26%) were aged 40–70 years. The vast majority of participants were non-Hispanics (851/1144, 89.79%). Among all participants, 951 (81.90%) experienced their first menstrual period after 11 years of age, 516 (36.87%) had 4 or more pregnancies, and 573 (45.74%) breastfed their children. The age at menopause of participants aged < 45 years, 46–55 years, and ≥ 56 years were 37.12, 53.1, and 9.11%, respectively. Approximately one-third of the participants had their final menstrual period 20 years previously, and half of the participants had a total reproductive lifespan of ≤ 35 years. The number of exogenous hormone users was as high as 783, accounting for 76.77% of all participants, which was more than three times the number of women who had never used exogenous hormones.

**Table 2:** Characteristics of study participants by distal sensory polyneuropathy. (NHANES 1999–2004 cycle, N = 1144).

Variables	Total	Non-DSP, n = 1055		DSP, n = 89		*p*-value
	n (%) ^a^	%^b^	(95% CI)	%^b^	(95% CI)	
Age						0.049*
40–70 years	798 (77.26)	94.71	92.57–96.39	5.29	3.61–7.43	
> 70 years	346 (22.74)	90.43	85.68–94.02	9.57	5.98–14.32	
Ethnicity						0.128
Hispanic	293 (10.21)	90.32	82.89–95.27	9.68	4.73–17.11	
Non-hispanic	851 (89.79)	94.13	92.28–95.65	5.87	4.35–7.72	
Education						0.013*
Less than high school	397 (22.93)	89.75	84.89–93.46	10.25	6.54–15.11	
High school and above	745 (76.97)	94.92	93.00–96.44	5.08	3.56–7.00	
Income status						0.139
PIR ≤ 2.00	436 (29.65)	91.95	87.52–95.19	8.05	4.81–12.48	
PIR > 2.00	592 (60.84)	95.16	92.62–97.02	4.84	2.98–7.38	
Insurance						0.669
Not covered	154 (10.33)	94.79	87.46–98.50	5.21	1.50–12.54	
Covered	979 (89.05)	93.69	91.92–95.16	6.31	4.84–8.08	
Alcohol use						0.233
Never	293 (22.03)	91.58	86.82–95.04	8.42	4.96–13.18	
Ever	850 (77.70)	94.33	92.00–96.14	5.67	3.86–8.00	
Smoking						0.849
Never	678 (55.84)	93.59	9138–95.38	6.41	4.62–8.62	
Ever	464 (44.09)	93.92	90.72–96.27	6.08	3.73–9.28	
Hypertension						0.759
No	435 (46.45)	93.3	89.55–96.02	6.70	3.98–10.45	
Yes	688 (51.70)	93.95	91.42–95.91	6.05	4.09–8.58	
BMI						0.370
Underweight/normal	356 (32.71)	95.12	91.65–97.45	4.88	2.55–8.35	
Overweight/obese	767 (65.86)	93.32	90.85–95.30	6.68	4.70–9.15	
Age at menarche						0.100
> 11 years	951 (81.90)	94.58	92.75–96.06	5.42	3.94–7.25	
≤ 11 years	179 (16.69)	91.04	84.98–95.24	8.96	4.76–15.02	
Gravidity						0.787
< 4	628 (63.13)	93.92	91.20–96.00	6.08	4.00–8.80	
≥ 4	516 (36.87)	93.43	90.75–95.53	6.57	4.47–9.25	
Breastfeeding history						0.197
Never	440 (41.34)	93.45	90.42–95.76	6.55	4.24–9.58	
Ever	573 (45.74)	95.45	93.13–97.16	4.55	2.84–6.87	
Age at menopause						0.083
≤ 45 years	409 (37.12)	93.58	90.54–95.88	6.42	4.12–9.46	
46–55 years	597 (53.17)	94.78	92.10–96.75	5.22	3.25–7.90	
≥ 56 years	133 ( 9.11)	87.94	79.01–94.02	12.06	5.98–20.99	
Time since menopause						0.049*
≤ 20 years	662 (66.08)	95.27	93.11–96.91	4.73	3.09–6.89	
> 20 years	411 (28.74)	92.28	89.17–94.72	7.72	5.28–10.83	
Total reproductive life						0.626
< = 35 years	558 (50.46)	94.06	91.56–96.01	5.94	3.99–8.44	
> 35 years	493 (41.90)	95.00	91.64–97.30	5.00	2.70–8.36	
History of exogenous hormone use						0.031*
Never	359 (22.83)	90.16	85.74–93.57	9.84	6.43–14.26	
Ever	783 (76.77)	94.77	92.61–96.45	5.23	3.55–7.39	

^a^Number (n) of participants with weighted column percentage (%). Column percentages for sample totals which do not add up to 100% are a result of missing data.

^b^Weighted row percentage with 95% confidence interval (CI). *DSP* Distal sensory polyneuropathy; *PIR* Prescribed investor rate; *BMI* Body mass index.

*p < 0.05 versus the values of non-DSP group.

### Distal sensory polyneuropathy and associated factors

The sociodemographic characteristics, health-related lifestyles, medical conditions, and reproductive factors of all participants were compared between the DSP and non-DSP groups (Table 3). Compared to the non-DSP group, women with DSP were more likely to be older (*p* = 0.049), have a lower education level (*p* = 0. 013), longer time since menopause (*p* = 0.049), and less likely to use exogenous hormones (*p* = 0.031).

**Table 3:** Multivariable logistic regression models assessing the association of reproductive factors and exogenous hormone use with distal sensory polyneuropathy (NHANES 1999–2004 cycle, n = 835).

Variables	Crude OR (95% CI)	*p*-value	Model 1		Model 2	
			Adjusted OR (95% CI)	*p*-value	Adjusted OR (95% CI)	*p*-value
Age at menarche						
≤ 11 versus (> 11 years)	1.72 (0.89, 3.30)	0.100	1.46 (0.54, 3.99)	0.447	8.13 (1.24, 53.28)	0.030*
Breastfeeding						
Ever versus (Never)	0.68 (0.38, 1.23)	0.197	0.41 (0.18, 0.93)	0.033*	0.45 (0.21, 0.99)	0.047*
Gravidity						
≥ 4 versus (< 4)	1.09 (0.59, 1.99)	0.270				
Age at menopause						
≤ 45 versus (46–55 years)	1.24 (0.63, 2.45)	0.520				
≥ 56 versus (46–55 years)	2.49 (1.10, 5.64)	0.030*				
Total reproductive life						
> 35 versus (< = 35 years)	0.83 (0.39, 1.76)	0.626				
Time since menopause						
> 20 versus (≤ 20 years)	1.69 (1.00, 2.85)	0.049*	3.01 (1.29, 7.02)	0.012*	3.18 (1.32, 7.68)	0.011*
History of exogenous hormone use						
Ever versus (Never)	0.51 (0.27, 0.94)	0.031*	0.48 (0.23, 1.00)	0.051	0.41 (0.19, 0.87)	0.022*

Values are odds ratio (OR) with 95% confidence interval (CI). Model 1 adjusted for age, ethnicity, education, income, body mass index (BMI); Model 2 adjusted for age, ethnicity, education, income, BMI, and interaction term of ethnicity with menarche (*p* for interaction = 0.027). Reference (OR = 1) for each variable was shown in the brackets.

*p < 0.05 versus the values of non-distal sensory polyneuropathy group.

### Association of reproductive factors and exogenous hormone with distal sensory polyneuropathy

Using a logistic regression model with purposeful selection of covariates, age, ethnicity, education, income status, BMI, age at menarche, breastfeeding history, time since menopause, and history of exogenous hormone use were included in the preliminary main effects model (Model 1 in Table 3, n = 835). In the interaction assessment with the preliminary main effects model, the two-way interaction term of menarche with ethnicity made contributed significantly to the fit of the model (*p* for interaction = 0.027); therefore, we added this interaction term as a source for confounding into the final model (Model 2 in Table 3, n = 835). After adjusting for all confounding factors, postmenopausal women with age at menarche ≤ 11 years exhibited a 8.13-fold higher risk of DSP than those with age at menarche > 11 years (OR = 8.13, 95% CI 1.24, − 53.28; *p* = 0.030); postmenopausal women with breastfeeding history experienced a 55% reduction in the odds of having DSP compared to those who breastfed no children (OR = 0.45, 95% CI 0.21, − 0.99; *p* = 0.047); postmenopausal women with time since menopause > 20 years were 3.18 times more likely to have DSP than those with shorter durations since menopause (< 20 years) (OR = 3.18, 95% CI 1.32, − 7.68; *p* = 0.011); and exogenous hormone users exhibited a reduction of 59% in the odds of having DSP compared to non-users (OR = 0.41, 95% CI 0.19, − 0.87; *p* = 0.022). Gravidity (crude OR = 1.09, 95% CI 0.59, − 1.99; *p* = 0.270) and total reproductive lifespan (crude OR = 0.83, 95% CI 0.39, − 1.76; *p* = 0.626) were not associated with DSP and were therefore not included in the final model. The age at menopause was not included in the final model. Age at menopause ≥ 56 years was associated with DSP (crude OR = 2.49, 95% CI 1.10, − 5.64; *p* = 0.030), but age at menopause < 45 years was not associated with DSP (crude OR = 1.24, 95% CI 0.63, 2.45; *p* = 0.520).

### Subgroup analysis by ethnicity

We conducted a subgroup analysis by ethnicity after finding the two-way interaction term of menarche with ethnicity in preliminary main effects model. According to the subgroup analysis, negative associations of older age at menarche (OR = 6.24, 95% CI 1.23, − 31.76; *p* = 0.028) and breastfeeding (OR = 0.22, 95% CI 0.06, − 0.84;* p* = 0.028) with DSP were observed among Hispanic women (n = 219). Among non-Hispanic women (n = 616), we found higher odds of DSP among women with time since menopause > 20 years (OR = 3.91, 95% CI 1.48, − 10.37; *p* = 0.007), and lower odds of DSP among exogenous hormone users (OR = 0.38, 95% CI 0.15, − 0.94; *p* = 0.036) (Table 4).

**Table 4:** Adjusted association of reproductive factors and exogenous hormone use with distal sensory polyneuropathy stratified by ethnicity (NHANES 1999–2004 cycle).

Variables	Hispanic, n = 219				Non-Hispanic, n = 616			
	Crude OR (95%CI)	*p*-value	Adjusted OR (95%CI)	*p*-value	Crude OR (95%CI)	*p*-value	Adjusted OR (95%CI)	*p*-value
Age at menarche								
≤ 11 versus (> 11 years)	3.41 (0.72,16.01)	0.102	6.24 (1.23, 31.76)	0.028*	1.37 (0.68, 2.77)	0.367	0.56 (0.15, 2.13)	0.384
Breastfeeding								
Ever versus (Never)	0.25 (0.07, 0.88)	0.024*	0.22 (0.06, 0.84)	0.028*	0.79 (0.39, 1.62)	0.520	0.61 (0.25, 1.47)	0.259
Time since menopause								
> 20 versus (≤ 20 years)	0.97 (0.24, 3.93)	0.959	1.98 (0.43, 9.04)	0.369	1.93 (1.13, 3.29)	0.016*	3.91 (1.48, 10.37)	0.007*
History of exogenous hormone use								
Ever versus (Never)	1.23 (0.35, 4.37)	0.740	0.50 (0.13, 1.97)	0.310	0.44 (0.22, 0.91)	0.025*	0.38 (0.15, 0.94)	0.036*

Values are odds ratio (OR) with 95% confidence interval (CI), after controlling age, education, income, body mass index (BMI). Reference (OR = 1) for each variable was shown in the brackets.

*p < 0.05 versus the values of non-distal sensory polyneuropathy group.

## Discussion

In this cross-sectional study of US postmenopausal women aged ≥ 40 years (NHANES 1999–2004), we observed negative associations of age at menarche, breastfeeding, and exogenous hormone use with DSP, and a positive association of time since menopause with DSP, after adjusting for important confounding variables. However, our study did not identify an association between gravidity, age at menopause, and total reproductive lifespan and DSP. We observed ethnicity differences in the associations between DSP and age at menarche, breastfeeding, time since menopause, and history of exogenous hormone use.

### Context in the literature

#### Age at menarche

Early menarche usually correlates with greater cumulative estrogen exposure among women and has been demonstrated to be a significant neuroprotective factor^12,29^. In contrast, we observed a higher risk of DSP among women with age at menarche ≤ 11 years. The negative effect of early menarche has been demonstrated in several health conditions, such as diabetes, metabolic syndrome, cardiovascular disease, and even mortality in previous studies^30,31^. The higher frequency of peripheral neuropathy in postmenopausal women with early menarche (≤ 11 years) may be explained by metabolic mechanisms. Women aged ≤ 11 years at menarche are more likely to have higher BMI or subcutaneous fat levels in childhood (5–9 years)^32^. Weight gain and increased fat may persist throughout adulthood among women with early menarche, which correlates with impaired insulin response, increased insulin resistance, and dyslipidemia, eventually resulting in a range of metabolic issues^33–35^. All these metabolic disorders resulting from early menarche might potentially increase the risk of DSP later in women’s lives^36,37^. The risk of metabolic syndrome decreases by 8% when age at menarche increases by one additional year^38^. Compared to women with late menarche (> 15 years), those with early menarche had a 1.62-fold higher risk of being affected by metabolic disorders^38^. In this study, we defined early menarche as age < 11 years and categorized all study participants into two groups. The effects of late menarche on DSP should be investigated in future studies. Future longitudinal studies should be conducted to observe menarche history, subcutaneous fat accumulation, BMI, insulin sensitivity, and the occurrence of DSP.

### Gravidity

Estrogen levels increase suddenly and dramatically (up to 300-fold) during early pregnancy and peak in late pregnancy, and higher gravidity might reflect a longer duration of exposure to high levels of estrogen^39^. However, we did not observe an association between gravidity and DSP among postmenopausal women in the US aged ≥ 40 years. Previous studies demonstrated a higher risk of peripheral nerve disorders during pregnancy and the postpartum period. These peripheral neuropathies are primarily caused by compression or weight gain, and their symptoms are usually temporary and reversible^40^. Pregnancy also has a complicated influence on metabolism, promoting increased insulin resistance, hyperlipidemia, adipogenesis and fat accumulation^41^. Frequent pregnancies have permanent adverse effects on lipid and glucose metabolism^42^. Our finding of the association between gravidity and DSP might be attenuated by aging and estrogen deficiency that occurs with menopause (natural ovarian failure or removal of ovaries)^43^. Besides the complex biological mechanisms, demographic and lifestyle factors might influence the association between gravidity and DSP. Women with greater pregnancies might have a better health condition because of a healthier lifestyle, better nutrition, and more social or family support from the multiparous family size^44–46^. Current evidence regarding the effect of gravidity on peripheral nerve conditions in postmenopausal women is limited. In our study, the number of gravidities was counted according to all types of pregnancies, including current pregnancy, live births, miscarriages, stillbirths, tubal pregnancies, or abortions. Future studies should examine whether pregnancy outcomes (such as live births) or pregnancy complications are associated with peripheral nerve changes.

### Breastfeeding

In our study, a lower risk of DSP was observed among postmenopausal women who breastfed their children. During breastfeeding, prolactin and oxytocin increase and become major driving hormones; however, estrogen and progesterone decrease rapidly^47^. Both prolactin and oxytocin are well-known neuroprotective factors that provide strong neuronal protection as neuropeptides by exerting anti-apoptotic, anti-inflammatory, and antioxidant properties^48,49^. Moreover, breastfeeding could help reverse the metabolic changes that occur during pregnancy, such as visceral fat and insulin resistance^43,50^. Several benefits of breastfeeding have been confirmed for maternal health, even in the absence of early, exclusive or continued breastfeeding^51^. Additionally, exclusive breastfeeding slows the return of ovulation after delivery and may slow depletion of the ovarian pool, which might consequently slow down reproductive aging^52^. Reproductive aging can influence the cellular, tissue, organ, and system aging of organisms^53^. Hence, reproductive and metabolism-related mechanisms may work together in providing the protective effect of breastfeeding on DSP. Future studies should examine the effects of breastfeeding duration on the peripheral nervous system.

### Menopause

Menopause marks the end of the reproductive phase of a woman's life, following a reduction in estrogen levels^54^. Postmenopausal status increases the risk of peripheral nerve disorders in non-diabetic obese women^16^. The age at menopause, total reproductive lifespan, and time since menopause were considered in this study. Early menopause is associated with shorter reproductive lifespan and lower cumulative estrogen exposure in women. Natural menopause usually occurs between the ages of 45–55 years worldwide^55^. The most prevalent type of premature or early menopause is surgical menopause, which causes abrupt loss of estrogen and progesterone in women^56^. In women with early menopause, shorter estrogen exposure can negatively affect the function of pancreatic β-cells and induce insulin resistance, which has demonstrated a harmful effect on peripheral nerves^57,58^. Additionally, early and premature menopause are more prevalent in women with chronic inflammatory diseases. Persistent chronic inflammation can induce peripheral neuropathy by impairing the efficiency of nerve regeneration and influencing the microenvironment in the peripheral nervous system, which is characterized by chronic macrophage infiltration, increased cytokine expression, and pro-inflammatory gene expression^59^. The term "total reproductive lifespan" refers to the time between menarche and menopause, which has been a superior variable for indicating the duration of exposure to endogenous estrogen, by comparing the age at menarche and age at menopause^13^. However, no associations between age at menopause and total reproductive lifespan with DSP were observed in our study.

Several factors may contribute to the absence of associations between distal sensory neuropathy and age at menopause and total reproductive lifespan. First, exogenous hormone use may influence these associations^60^. Second, surgical and natural menopause have inconsistent effects on women's health outcomes^56^. Premature or early natural menopause is usually a gradual process. In contrast, surgical removal of both ovaries causes an abrupt loss of ovarian hormones, including estrogen, progesterone, and testosterone, as well as damage to the hypothalamus–pituitary–gonadal axis^56^. However, our study did not differentiate between natural and surgical menopause in this regard. The characteristics of the study population should be considered in detail in future studies when exploring the associations between age at menopause and total reproductive lifespan with DSP.

Time since menopause > 20 years was found to be an independent risk factor for DSP among postmenopausal women aged ≥ 40 years in our study. In contrast to age at menopause and total reproductive lifespan (correlates to exposure to endogenous estrogen during the premenopausal phase), time since menopause could be used as a marker of endogenous estrogen deficiency during the postmenopausal phase. A longer time since menopause impairs glucose tolerance among women, which could induce neuropathies, mostly on the small nerve fibers^61,62^. Previous studies have demonstrated that women at > 20 years since menopause are at a greater risk of metabolic syndrome, cardiovascular disease, and elevated blood pressure than those at < 10 years since menopause^63^. In addition, the longer time since menopause could accelerate biological aging compared to a shorter duration after menopause, which might also accelerate peripheral nerve impairment^64^.

### Exogenous hormone use

In our study, exogenous hormones are referred to as menopausal hormone therapy or contraceptive use, including exogenous estrogen and progesterone, both of which are neuroprotective factors^19,65^. Although both estrogen and progesterone are well-known neuroprotective factors, there is currently limited research on whether a history of exogenous hormone use affects peripheral nerves. We found that exogenous hormone users experienced a lower risk of DSP among postmenopausal women aged ≥ 40 years. In previous animal studies, aging nerves remained sensitive to estrogen and progesterone^19^. Estrogen and progesterone can regulate the expression of nerve growth factors in the peripheral organs of ovariectomized female mice. Nerve growth factor is a neurotrophic factor known to play a key protective role in the development and survival of sympathetic, sensory, and forebrain cholinergic neurons^19^. The influence of exogenous hormones on postmenopausal female neurological systems is currently under investigation. These contradictory results might be related to differences in hormone formulation and use, characteristics of hormone users, and variety of nerve types^66^. The regimen, timing, and dose of the exogenous hormones should be considered in future studies.

### Ethnicity differences

It is important to conduct the racial/ethnic difference studies for identifying and addressing health disparities, understanding the related root causes^67^. However, it also necessary to be cautious for the distinctions between race and ethnicity because they are complex and multifaceted constructs^67^. In our study, we collapsed Mexican American and other Hispanic as Hispanic, while Non-Hispanic White, Black and Others as Non-Hispanic, because the small sample size from each race group prevented us from analyzing this issue via subgroup analysis. Hispanics are the largest minority group in the United States, who are exposed to higher health risks^68^. Female Hispanics are a growing population group and are exposed to poor social and health circumstances^68^. In the subgroup analysis, the associations of age at menarche and breastfeeding with DSP were only observed in the Hispanic group. In the US, the average age at menarche was > 14 years before 1900, but decreased to 12.43 years in 1988–1994 and 11.9 years in 2013–2017^69–71^. Mexican Americans, the majority of the US Hispanic population, had the fastest rate of decline in the age of menarche^72^. Obesity is a major health problem in Hispanics^68^. Overweight or obese women usually have more metabolic disorders and inflammation^2,73^. Therefore, the results of the subgroup analysis suggest that the effect of age at menarche and breastfeeding on peripheral nerves might be closely associated with metabolic and inflammatory issues.

We identified a significant positive association between the time since menopause and exogenous hormone use with DSP only in the non-Hispanic group. The association between the time since menopause and DSP might be explained by estrogen levels^74^. Estrogen levels in postmenopausal women reach their lowest point in the first 2 years after the final menstrual period^54^. Estrogen levels during the postmenopausal phase in non-Hispanic women are lower than those in Hispanics. Moreover, Hispanic women usually have the highest rates of obesity^68^. Adipocytes produce estrogens through aromatase activity, and obese women have higher levels of circulating estrogens than other women during the postmenopausal phase^75^. Hence, ethnicity differences in estrogen levels during the postmenopausal phase and obesity status might potentially lead to ethnicity heterogeneity in the association between time since menopause and DSP. In addition, Hispanics in the US are covered by fewer preventive health services than other ethnic groups^68^. This could be a potential explanation for the absence of an association between exogenous hormone use and DSP.

### Strengths and limitations

This study has several strengths. First, we investigated the association between reproductive factors and DSP in postmenopausal women using nationally representative data. Second, we used logistic regression with a purposeful selection strategy to assess the association between exposure and outcome. This strategy allowed us to correctly identify and retain confounders at a higher rate than other selection algorithms. Our findings might provide a rationale for the occurrence and development of DSP in women. Finally, we identified that the effects of reproductive factors and exogenous hormone use on DSP were not constant by ethnicity.

Despite its strengths, this study has several limitations. First, the assessment of peripheral neuropathy was limited to the monofilament test which was the only available test in NHANES 1999–2004. Therefore, we cannot conclude whether our findings from a small number of events could be generalized among current postmenopausal women in the because NHANES 1999–2004 was conducted almost 20 years ago. Additonally, other objective examinations, such as nerve conduction studies or skin biopsies, were not available from the NHANES. Second, bias should be considered. Recall bias in assessing exposure may have existed among postmenopausal women in this study, especially among those of advanced age. A selection bias from excluding those with medical diseases may have led to an apparent association. Third, additional study participants’ characteristics based on blood tests were not provided in our study, because the sample size in the fasting subsample was small. Our study did not include blood cholesterol or other inflammatory biochemical markers as potential confounders, which may explain the underlying mechanisms of DSP. Diabetes was defined as a self-reported medical history through interviews. Therefore, non-diabetic status may be misclassified. Fourth, premature or early natural menopause is usually a gradual process, while surgical removal of both ovaries causes an abrupt loss of ovarian hormones. The natural menopause or surgical menopause should be distinguished in the future study. Fifth, this was a cross-sectional study, and only the prevalence of DSP was reported; therefore, a causal relationship could not be confirmed. Lastly, the study population included only postmenopausal women in the United States. Future studies should be conducted among participants in different countries and ethnic groups and ethnicity differences should be considered.

## Conclusion

Age at menarche, time since menopause, breastfeeding, and exogenous hormone use were associated with DSP among postmenopausal US women aged ≥ 40 years. Ethnicity-based heterogeneity was also observed in these associations. Our findings may provide a rationale for the etiology of peripheral neuropathy among postmenopausal women in the United States. Reproductive factors may influence peripheral nerve health via both hormone- and metabolism-related pathways. Greater exposure to estrogen before menopause might not prevent peripheral neuropathy. In contrast, estrogen deficiency might be associated with biological aging to cause peripheral nerve damage in postmenopausal women. Therefore, promoting breastfeeding may reduce the burden of peripheral neuropathy in middle-aged postmenopausal women. Exogenous hormone use may help slow nerve damage or recover damaged nerves, and could potentially be used in the management of peripheral neuropathy. Further research is required to investigate the underlying mechanisms.

## Supplementary Information

Supplementary Information.

## Data Availability

Data from NHANES could be downloaded online. The datasets generated and analyzed during the current study are available from the first author.
