# Supplementary material for: Association of reproductive factors and exogenous hormone use with distal sensory polyneuropathy among postmenopausal women in the United States: results from 1999 to 2004 NHANES
Source: Sci Rep. 2023 Jun 7;13:9274. doi: 10.1038/s41598-023-35934-7 (PMC10247787; doi:10.1038/s41598-023-35934-7)
Supplement: Supplementary file 1 — Supplementary Information. [file 41598_2023_35934_MOESM1_ESM.docx]

**Supplemental Table S1. Codebook**

| **Variables (labels)** | **Code** | **Value Description** | **Count** | **Missing (%)** |
| --- | --- | --- | --- | --- |
| **Dependent variable** | | | | |
| PNEXAM | 0 | Non-DSP | 1,055 |  |
| DSP | 1 | DSP | 89 |  |
|  | . | missing | 0 | 0 |
| **Independent variables** | | | | |
| MENARCHE | 0 | > 12 y | 951 |  |
| Age at menarche | 1 | ≤ 11 y | 179 |  |
|  | . | missing | 14 | 1.22 |
| PREGNANT | 0 | <4 | 525 |  |
| Number of pregnant | 1 | ≥4 | 516 |  |
|  | . | missing | 103 | 9 |
| BREASTFED | 0 | Never | 440 |  |
| Breastfed any of your children? | 1 | Ever | 573 |  |
|  | . | missing | 131 | 11.45 |
| AGEATMENO | 1 | <45 y | 409 |  |
| Age at menopause | 2 | 46-55 y | 597 |  |
|  | 3 | ≥56 y | 133 |  |
|  | . | missing | 5 | 0.44 |
| TSMENO | 0 | ≤20 y | 662 |  |
| Time since menopause | 1 | > 20 y | 411 |  |
|  | . | missing | 71 | 6.21 |
| REPROLIFE | 0 | <=35 y | 558 |  |
| Total reproductive life | 1 | >35 y | 493 |  |
|  | . | missing | 93 | 8.13 |
| HORMONE | 0 | Never | 359 |  |
| History of exogenous hormone use | 1 | Ever | 783 |  |
|  | . | missing | 2 | 0.17 |
| **Confounding variables** | | | | |
| AGEGROUP |  |  |  |  |
| Age at Screening | 0 | 40-70 y | 798 |  |
|  | 1 | ≥ 70 y | 346 |  |
|  | . | missing | 0 | 0 |
| RIDRETH1 | 0 | Hispanic | 293 |  |
| Race/Ethnicity | 1 | Non-Hispanic | 851 |  |
|  | . | missing | 0 | 0 |
| DMDEDUC | 0 | Less Than High School | 397 |  |
| Education | 1 | High School and over | 745 |  |
|  | . | missing | 2 | 0.17 |
| INCOME | 0 | PIR ≤ 2.00 | 436 |  |
| Family income with PIR | 1 | PIR > 2.00 | 592 |  |
|  | . | missing | 116 | 10.14 |
| ALCOHOL | 0 | Never | 293 |  |
| Alcohol use | 1 | Ever | 850 |  |
|  | . | missing | 1 | 0.09 |
| SMOKING | 0 | Never | 678 |  |
| Smoking status | 1 | Ever | 464 |  |
|  | . | Missing | 2 | 0.17 |
| HYPERTENSION | 0 | No | 435 |  |
| Hypertension | 1 | Yes | 688 |  |
|  | . | missing | 21 | 1.84 |
| BMIGRP | 0 | Underweight/normal | 356 |  |
| BMI | 1 | Overweight/Obese | 767 |  |
|  | . | missing | 21 | 1.84 |
| INSURANCE | 0 | Not covered | 154 |  |
| Insurance | 1 | Covered | 979 |  |
|  | . | missing | 11 | 0.96 |
